# Case report of cavernous hemangioma with a 4 year follow up

**DOI:** 10.1016/j.amsu.2022.104027

**Published:** 2022-06-19

**Authors:** Oufaa Jamal, Saad Hmada, Khalid Aadoud, Iatimad Elbejjaj, Mehdi Elkarkouri, Abderrazak Bertal, Abdelhakim Lakhdar

**Affiliations:** Department, University Hospital Center IBN ROCHD, Casablanca, Morocco

**Keywords:** Cavernous hemangioma, Spine, Epidural, Case report

## Abstract

Cavernous hemangiomas are vascular malformations that can affect any part of the central nervous system. In general, epidural hemangiomas are secondary extensions of spinal lesions. These tumors grow slowly and are expressed as slow spinal cord compression syndromes, radiculopathy, or both. History, clinical examination, routine radiographs, MRI, and histopathologic studies are aids to a definitive diagnosis. This is a 61-year-old chronic smoker with a history of cholecystectomy in 2017. History of the disease: dates to 1 month by a progressive installation of heaviness of the left lower limb, then of the right one 15 days later. The state was complicated one week before his admission by sphincter disorders such as urinary leakage. Clinically, the patient walked with assistance, with a paraparesis of the two lower limbs at 4/5 on muscle testing, with a posterior cord syndrome, a D6 sensory level and normal osteotendinous reflexes. Spinal cord MRI showed a tissue-like process at D6-D7 extra-medullary extradural. Spinal cavernous extradural hemangioma is a frequent lesion, represented by a malformation of the microcirculation, whose diagnosis has become easier with the advent of MRI, revealed essentially by a spinal cord compression syndrome, whose evolution is favorable if treated in time.

## Highlights

• This article presents a case of a dorsal epidural cavernous hemangioma. The clinical examination revealed a posterior cord syndrome associated with sphincter disorders such as urinary leakage. The medullary MRI confirmed the suspected diagnosis of medullary compression.
• The total removal of the tumor was done, the diagnosis of cavernous hemangioma was suspected intraoperatively, and confirmed in anatomopathological examination.
• The follow up done up to 4 years showed the complete recovery of the patient showing the importance of time-based care.

## 1 Introduction

Cavernous hemangiomas are vascular malformations that can involve any part of the central nervous system. In general, epidural hemangiomas are secondary extensions of spinal lesions. These tumors grow slowly and are expressed as slow spinal cord compression syndromes, radiculopathy, or both. History, clinical examination, routine radiographs, MRI, and histopathologic studies are aids to a definitive diagnosis.

## 2 Case report

This is a 61-year-old chronic smoker with a history of cholecystectomy in 2017. History of the disease: dates to 1 month by a progressive installation of heaviness of the left lower limb then of the right one 15 days later. His case was complicated one week before his admission by sphincter disorders such as urinary leakage.

Clinically, the patient presented a paraparesis with a score of 4/5 and a posterior cord syndrome with cautious walking with assistance and a D6 sensory level and normal osteotendinous reflexes.

**Fig. 1:**
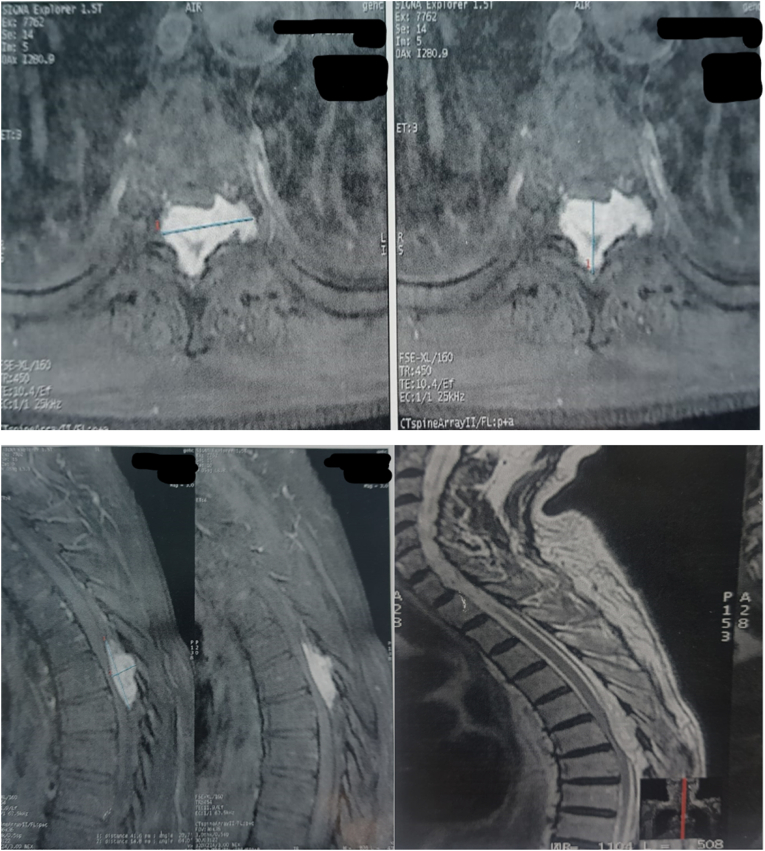
Spinal cord MRI revealed a tissue-like process at the level of T6-T7, intradural, intermediate T1 signal and T2 hypersignal and strongly enhanced after gadolinium injection with left foraminal extension measuring 25 mm in width and 13.5 mm in anteroposterior diameter and extending over a height of 30 mm.

A radiological examination of thoracolumbar spine has been performed (Fig. 1).

**Fig. 2:**
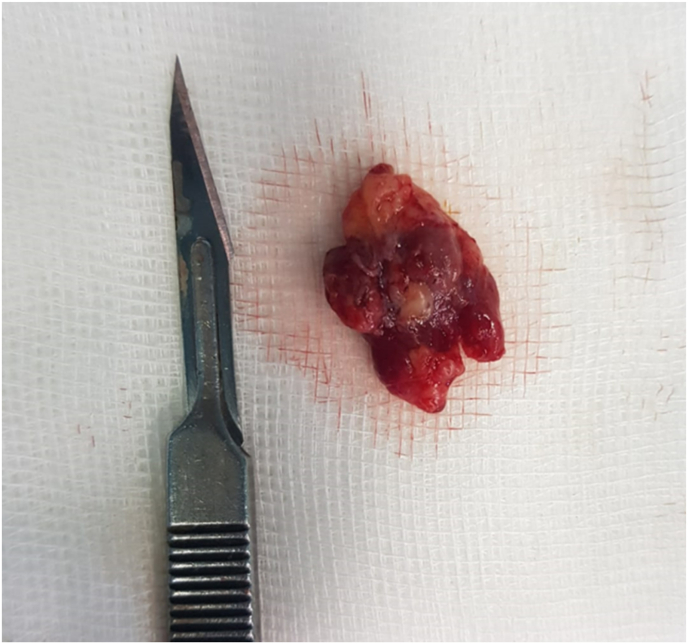
Image showing the size of the tumor compared to a number 11 scalpel blade.

We made the surgical indication. The surgical intervention was carried out by our professor during which we performed a laminectomy of D6-D7, we discovered a reddish intra canal epidural process with a feeding pedicle from which we proceeded to the total removal (Fig. 2).

The post-operative course was simple, the surgical wound was clean. Neurologically, the patient remained stationary, walking with a cane.

**Fig. 3:**
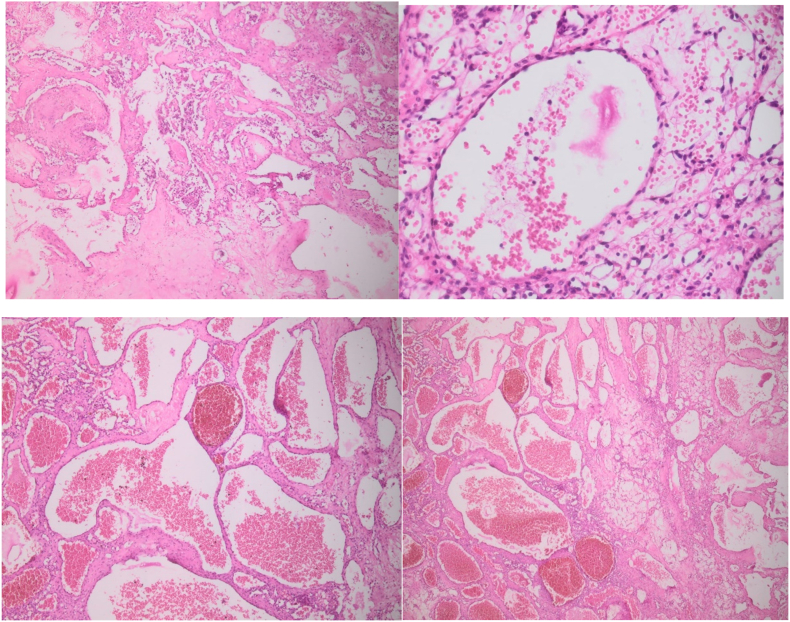
Electron microscope images of the slides of the tumor product we found.

The anatomical pathological examination was in favor of an extra-dural cavernous hemangioma (Fig. 3).

**Fig. 4:**
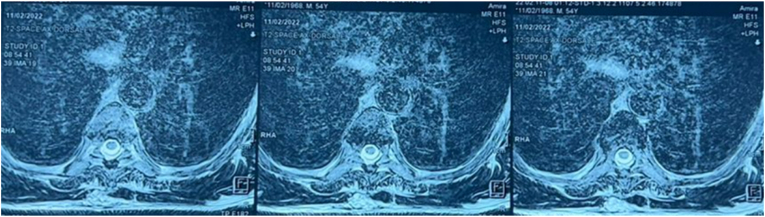
The follow-up MRI performed at 2 years postoperatively did not show any recurrence.

The evolution at 1 year is marked by the improvement of the clinical picture, in particular walking without helps and regression of the posterior cord syndrome. The six-monthly monitoring o objectified the complete improvement of his clinical picture. Our monitoring up to 4 years of age has shown a stability of his clinical condition including normal unaided walking and no sphincter disorders (Fig. 4).

This case has been reported in line with the 2020 SCARE guidelines [9].

## 3 Discussion

Cavernous angiomas of the spine are benign and are vascular hamartomas representing a dysplasia of the vessel-forming mesoderm. Harrison and al. showed that primordial vessels may lose their ability of differentiation, resulting in a cavernous malformation [3].

Vertebral cavernous angiomas (VCAs) are either capillary or cavernous in histology, and sometimes mixed. They are frequent (more than 10% of the elderly population), asymptomatic and purely spinal. They predominate in the middle and lower dorsal region.

Extradural spinal cavernous hemangiomas represent 4% of spinal epidural lesions. This location is often secondary to an epidural extension of a vertebral hemangioma [1].

A review of literature done by GIRISH krichna and al that is showing thoracic spinal cord was the most common location. In 27 cases, thoracic spinal cord was involved. The cervical spine was involved in 6 cases. The lumbar spine was involved in 11 cases. The sacral epidural space was involved in 1 case, which is concordant with our case, and showing the predominance of this localization [2].

Cavernous angiomas do not grow by mitotic activity but have the propensity to enlarge by thrombosis and bleeding, causing a spectrum of neurological syndromes ranging from radiculopathy to sudden spinal cord dysfunction [5], These tumors grow slowly and produce symptoms of progressive myelopathy, radiculopathy, or both [8].

MRI finding showed an epidural lesion of different sizes. It is hyperintense on T2-weighted image and isointense on T1-weighted image but is enhanced after injection of gadolinium in a very intense and homogeneous way. The lesion was completely extradural but tightly adherent to the dura mater. the surgical excision is not too hemorrhagic.

From the surgical point of view, it is very important to understand that the extra-axial cavernous angiomas behave like tumors and not like vascular malformations [6]. Surgical decompression is the main treatment. Adjuvant radiotherapy has no place in first line but has been used in 2 cases where the symptomatology remained unchanged or worsened [2].

Microscopically, cavernous malformations are composed of closely opposed sinusoidal vascular spaces. The walls consist of an innermost single layer of endothelial cells surrounded by adipose tissue; elastic fibers or smooth muscle cells are absent [4], [7].

## 4 Conclusion

Cavernous angiomas of the spine are benign and are vascular hamartomas, despite their vascular nature, they behave like tumors, spinal cord MRI is the key examination, and surgery is the treatment of choice. If managed in time, the neurological evolution is generally favorable without the need for additional treatment.

## Ethical approval

Written informed consent was obtained from the patient for publication of this case report and accompanying images. A copy of the written consent is available for review by the Editor-in-Chief of this journal on request.

## Sources of funding

None.

## Author contribution

Oufaa JAMAL: writing the paper, Saad HMADA: Corresponding author writing the paper, Khalid AADOUD: study concept, Iatimad ELBEJJAJ: study concept, Abderrazak BERTAL: Correcting the paper, Mehdi ELKARKOURI: Correcting the paper, Abdessamad NAJA: Correcting the paper, Abdelhakim LAKHDAR: Correcting the paper.

## Registration of research studies

Name of the registry: xxxxx

Unique Identifying number or registration ID: xxxx

Hyperlink to your specific registration (must be publicly accessible and will be checked): xxxx

## Guarantor

Saad HMADA, Oufaa JAMAL.

## Consent

The consent has been done and is available on request, also the manuscript respects the privacy of the patient.

## Provenance and peer review

Provenance and peer reviewNot commissioned, externally peer-reviewed.

## Declaration of competing interest

The authors of this article have no conflict or competing interests. All the authors approved the final version of the manuscript.
